# INTERVENTIONS FOR LONELINESS AMONG OLDER ADULTS IN FACILITIES: A SYSTEMATIC REVIEW

**DOI:** 10.1093/geroni/igz038.2884

**Published:** 2019-11-08

**Authors:** Nicolas Quan, Matthew Lohman

**Affiliations:** University of South Carolina, Columbia, South Carolina, United States

## Abstract

Loneliness affects an estimated 1 in 3 older adults ages 65 and over in America. According to a 2017 report by AARP, a lack of meaningful social contact among older adults is associated with $6.7 billion dollars of federal healthcare spending. Research suggests that interventions such as social facilitation and skills development may decrease loneliness; however, the effectiveness of such interventions for older adults living in long-term care facilities is unclear. Articles matching search criteria were collected from PubMed, PsycInfo and Web of Science from 2009 to 2019. Inclusion criteria were: 1) intervention studies, 2) individuals age >= 65, 3) participants living in a long-term care facility such as a nursing home, assisted-living, or hospice facility. Randomized controlled trials, quasi-experimental and single-group studies were included. Title and abstract screening, as well as full text extraction followed PRISMA guidelines. A total of 16 articles that met inclusion criteria were identified. The interventions included video chatting with family members, human-volunteer interaction, human-robot interaction, humor therapy, a reminiscent radio program, laughter therapy and gardening education. Fourteen studies demonstrated a statistically significant decrease in loneliness from baseline to post-intervention. Laughter therapy showed the greatest reduction in loneliness. Diversity of intervention types and loneliness measures meant we could not estimate a pooled measure of effectiveness. Results suggest that there are several effective interventions to reduce loneliness among older adults in facilities; however, lack of standardized measures and high-quality studies limits comparisons between intervention types and generalizability to different populations.

